# Awareness of Lifestyle Modifications in the Management of Polycystic Ovarian Syndrome: A Hospital-Based Descriptive Cross-Sectional Study

**DOI:** 10.7759/cureus.36889

**Published:** 2023-03-29

**Authors:** Asmita Kaundal, Prachi Renjhen, Rajeshwari Kumari

**Affiliations:** 1 Obstetrics and Gynaecology, All India Institute of Medical Sciences, Bilaspur, Bilaspur, IND; 2 Obstetrics and Gynaecology, Dr. Baba Saheb Ambedkar Medical College and Hospital, Delhi, IND

**Keywords:** polycystic ovary syndrome, adolescent pcos, knowledge attitude, lifestyle behaviour, pcos and metabolic syndrome

## Abstract

Objective: Polycystic ovarian syndrome (PCOS) is a prevalent endocrinological disorder in reproductive-age women. Due to varied presentations, it's often difficult to diagnose and manage women with PCOS. Management usually focuses on treating the symptoms and preventing long-term sequelae of the disease. This study was planned to assess the knowledge among reproductive-age women (15-44 years) regarding the risk factors, symptoms, complications, and management of PCOS.

Material and methods: This is a hospital-based descriptive cross-sectional study. A pre-validated well-structured questionnaire which included basic demographic data, menstrual history, knowledge about PCOS symptoms, risk factors, complications, prevention, and treatment, was administered. Completed questionnaires were analyzed to calculate the knowledge score of the participants and its association with their education level and occupation was seen.

Results: A total of 350 women participated but only 334 completed questionnaires were included for final evaluation. The mean age of the study population was 28.70±6.29 years. Around 9.3% of the participants were already diagnosed with PCOS. Most of the women (43.4%) had heard about PCOS. The source of information was doctors (26.6%), the internet (6.28%), teachers (5.6%), and friends (4.7%). Obesity (33.5%), unhealthy dietary habits (35%), and genetic predisposition (40.7%) were thought as risk factors for PCOS. Most of the participants were aware that subfertility (40.1%), abortions (34.4%), diabetes (28.7%), hypertension (31.7%), cardiovascular disease (33.5%), endometrial carcinoma (35.9%), and psychological disturbances (37.1%) are among the known PCOS related complications. Eating a healthy diet (37.1%) and weight reduction (41%) can help in the management of PCOS. Around 60.5% of women showed poor knowledge, 14.7% fair knowledge, and 24.9% good knowledge regarding PCOS. Education level and occupation status were found to be significantly related to the knowledge score (P≤0.001).

Conclusion: PCOS is a prevalent condition with varied presentations which significantly affects one's quality of life. Since there is no definitive treatment for PCOS the management generally aims at managing symptoms and reducing the risk of long-term complications. To reduce the burden of PCOS-related long-term complications behavioral changes in terms of regular exercise and healthy dietary habits need to be incorporated from childhood.

## Introduction

Polycystic ovarian syndrome (PCOS) has emerged as a new epidemic in the last few decades. The prevalence of PCOS varies between 6% and 25% globally [1-6]. Due to the complex presentation of the disease diagnosis is difficult and is often delayed [7-9]. Women with PCOS may present with menstrual irregularities, subfertility, obesity, and dermatological manifestations like hirsutism and acne. The long-term sequelae of the disorder include metabolic disorders, impaired glucose tolerance, diabetes, hypertension, and cardiovascular disorders. Due to chronic anovulation and the unopposed effect of estrogen on the uterus, these women are at higher risk of developing endometrial cancer later in life. Adolescent women with PCOS may suffer from various psychological issues like depression, anxiety, sleep disturbances, and body image disorders which significantly affect their quality of life. The exact etiology of the disorder is not known however oxidative stress, genetic predisposition, and certain gene polymorphisms are thought to be the culprit for PCOS [10-14]. Various environmental factors, sedentary lifestyles, and unhealthy eating habits are also seen to be related. Rotterdam criteria, National Institute of Health (NIH), and androgen excess-PCOS Society criteria are used to diagnose the disorder. The management is usually targeted to treat the symptoms and prevent long-term sequelae. Lifestyle modifications in terms of adopting an active lifestyle, healthy and balanced diet, avoiding junk and unhealthy eating habits, weight reduction in those who are overweight and stress management have been seen to be effective. Even in those who need pharmacotherapy coupling drug therapy with lifestyle modification has shown better results [15,16].

This study was planned to assess the level of knowledge among the reproductive age woman about PCOS, risk factors and complications, and the source of information about the disease so that strategies can be planned to disseminate awareness regarding PCOS at the community level.

This article was previously posted to the research square preprint server on 25th February 2022.

## Materials and methods

A hospital-based descriptive cross-sectional study was done in reproductive-age women attending the outpatient department of Gynaecology at a tertiary care hospital. After obtaining ethical clearance from the ethical committee of the institute, a pre-validated well-structured questionnaire containing 40 items was administered by the interviewers after explaining the purpose of the study and obtaining informed consent. All the women between the age group of 15-50 years who attended the Gynecology OPD of the hospital and were able to understand either Hindi or English language were invited to fill out the questionnaire. For those who could not read or write but consented to participate in the study, the questionnaire was read out to them by the investigator and answers were marked as per the response by the participants. A total of 350 women participated. Out of all around 16 forms were incomplete so were not included in the final analysis. Demographic data (Item no. 1-7), information related to the menstrual history (Item no. 8-11), presence of any symptom of PCOS (Item no. 9-18), information regarding knowledge about PCOS, source of information, its symptoms and complications related to it (Item no. 19-30), regarding their knowledge about the prevention and treatment of the PCOS related symptoms and complication (Item no. 31-36) were collected. Information was also collected about the healthy lifestyle activities being practiced by the study cohort (Item no. 37-40).

Statistical analysis

In the present study, descriptive and inferential statistical analysis has been carried out. Results on continuous measurements are presented as mean ± SD (Min-Max). Results on categorical measurements are presented as numbers (%). Significance is assessed at a 5% level of significance. The one-way analysis of variance (ANOVA) is employed to determine whether there are any statistically significant differences between the means of three or more independent (unrelated) groups. The significance of the parameters on the categorical scale between two or more groups has been calculated using Chi-square/Fisher Exact test. The non-parametric setting was used for qualitative data analysis. For very small cell samples Fisher Exact test is used. P-value: 0.05<P<0.10 was taken as suggestive significant, P-value: 0.01<P<0.05 as moderately significant, and P-value: P<0.01 as strongly significant. Statistical software namely Statistical Product and Service Solutions (SPSS) (IBM SPSS Statistics for Windows, Version 22.0, Armonk, NY), and R environment ver. 3.2.2 were used for the analysis of the data, and Microsoft Word and Excel have been used to generate graphs, tables, etc.

## Results

The demographic data of the study participants are depicted in Table 1. The mean age of the study population was 28.70±6.29 years. Most of the women in the study group were between 21 and 30 years (61.4%). The majority of the study participants had normal BMI (49.4%). Only 13.5% were underweight (<18.5), and around 28.7% were overweight (25.0-29.9). Around 8.4% of participants were obese (>30). Most of the women in the study group were graduates (42.8%). Most of them were housewives (49.4%) and more than half of the study participants were married (61.7%).

**Table 1:** Demographic Profile of Study Population

Variable	N=334	% age	Mean ± SD
Age (in years)			28.70±6.29
15-20	09	2.7%	
21-30	205	61.4%	
31-40	102	30.5%	
41-50	18	5.4%	
BMI			23.61±4.54
<18.5	45	13.5%	
18.5-24.9	165	49.4%	
25.0-29.9	96	28.7%	
>30.0	28	8.4%	
Education			
Illiterate	62	18.6%	
Primary	27	8.1%	
High School	74	22.2%	
Graduate	143	42.8%	
Postgraduate	28	8.4%	
Occupation			
Housewives	165	49.4%	
Working (private/Government/Self)	79	23.6%	
Students	90	26.9%	
Marital Status			
Married	206	61.7%	
Unmarried	128	38.3%	
Age at Menarche			
<13 years	24	7.2%	13.48±1.00
13-15 years	271	81.1%	
ears	31	9.3%	
>18 years	none	none	
Cycle Length			21.56±3.28SD
<21 days	30	8.9%	
21-35 days	215	64.4%	
35-60 days	78	23.4%	
>60 days	05	1.5%	
Totally invariable	06	1.8%	

The mean age of menarche in the study cohort was found to be 13.48±1 years. The mean cycle length was 21.56+3.28 days with cycle lengths of 21-35 days in 64.37%, <21 days in 8.9%, 35-60 days in 23.35%, and >60 days in 1.49%.

Table 2 shows the distribution of symptoms related to PCOS among the study participants. Among all 35.6% of the participants experienced a change in the cycle length in the past six months. Around 9.0% said they were having frequent cycles (<21 days), 24.8% reported delayed cycles (>35 days), 1.5% reported scanty flow, and 0.3% were having heavy flow during the cycle. Out of all 9.8% of the participants had taken medication for the regularization of their menstrual cycles. Among the study participants, 6.3% experienced weight gain, and 1.2% weight loss in the past six months. However, the majority experienced no change in their weight in the previous six months. Around 2.4% of women experienced excessive hair growth on the body, 9.0% said they were having excessive hair fall, and 8.7% reported acne and needed treatment. Around 8.1% of the women were desirous of pregnancy out of which 6.6% were having some difficulty in spontaneous conception and 4.4% were already on treatment for subfertility. In the present study, 9.3% of the participant were already diagnosed to have PCOS.

**Table 2:** Presence of Symptoms Related to PCOS PCOS: polycystic ovarian syndrome

S.No.	Symptoms Present	N=334	% age
1.	Change in cycle length in previous 6 months		
	Yes	119	35.6%
	No	215	64.4%
2.	What change		
	Frequent Cycle	30	9.0%
	Delayed Cycles	83	24.8%
	Heavy Cycles	1	0.3%
	Scanty Cycles	5	1.5%
3.	Treatment taken to correct menstrual irregularity in past 6 months		
	Yes	33	9.8%
	No	301	90.1%
4.	Change in weight in previous 6 months		
	Yes	25	7.5%
	No	307	91.9%
5.	What Change		
	Weight Gain	21	6.3%
	Weight Loss	4	1.2%
	No Change	307	91.9%
6.	Excessive hair growth on body in past 6 months		
	Yes	8	2.4%
	No	326	97.6%
7.	Excessive hair fall		
	Yes	30	9.0%
	No	304	91.0%
8.	Acne		
	Yes	29	8.7%
	No	305	91.3%
9.	Are you trying to conceive		
	Yes	27	8.1%
	No	307	91.9%
10.	Any problems in conception		
	Yes	22	6.6%
	No	312	93.4%
11.	Taking any fertility treatment	15	4.4%

Table 3 depicts the knowledge of study participants regarding PCOS. It was seen that only 43.4% of the women had heard about PCOS and the most common source of their information was doctors (26.6%) followed by the internet (6.28%), teachers (5.6%), and friends (4.7%). Assessment of knowledge for risk factors showed that out of those who had heard of PCOS, a significant proportion of women were aware of the association of obesity (33.5%), unhealthy dietary habits (35%), and genetic predisposition (40.7%) with PCOS. When asked about complications and long-term sequelae of PCOS 40.1% of women said that patients with PCOS can have problems in conception, 34.4% said it PCOS women are at higher risk of pregnancy loss, 28.7% said women with PCOS are prone to develop diabetes, 31.7% said they can have hypertension, 33.5% said it can raise the chances of cardiovascular disease, 35.9% said there could be an association with endometrial carcinoma and 37.1% said it could be related to psychological problems, sleep disturbances, body image disorder, and anxiety in women. Around 37.1% were aware that eating a healthy and balanced diet can be one of the management methods, 41% believed weight reduction could help, and 35.6% said trying ways to reduce stress could be one of the methods to manage the stress and mood-related symptoms in PCOS women. However, 34.1% said only medications can treat the condition and 2.7% believed only surgery can treat PCOS.

**Table 3:** Awareness Regarding Symptoms, Risk Factors, Complications, and Management of PCOS PCOS: polycystic ovarian syndrome

Question	N=334	% age
Heard about PCOS		
Yes	145	43.4%
No	189	56.6%
Have you been diagnosed as PCOS		
Yes	31	9.3%
No	303	90.7%
Source of Information	N=145	
Teacher	19	5.6%
Doctor	89	26.6%
Friend	16	4.7%
Television	0	0%
Newspaper	0	0%
Internet	21	6.3%
Symptoms: PCOS can manifest as	N=145	
Menstrual Irregularity	142	42.5%
Hirsutism	90	26.9%
Acne	70	20.9%
Weight gain	119	35.6%
Difficulty in conception	117	35.02%
Abortions	115	34.4%
Hair fall	82	24.8%
Risk Factor	N=145	
Obesity	112	33.5%
Unhealthy eating habits	117	35%
Sedentary lifestyle	121	36.2%
Genetic	136	40.7%
Complications	N=145	
Infertility	134	40.1%
Abortions	115	34.4%
Diabetes	96	28.7%
Hypertension	106	31.7%
Cardiovascular Disorders	112	33.5%
Endometrial Carcinoma	120	35.9%
Psychological problem, anxiety, sleep disturbances	124	37.1%
Management	N=145	
Avoiding junk food and healthy eating habits	124	37.1%
Exercise and weight loss	137	41%
Stress management	119	35.6%
Medications	114	34.1%
Surgery	96	2.7%

A total of 14 items were used to assess the knowledge regarding risk factors, symptoms, long-term complications, prevention, and treatment of PCOS. For every correct answer participants were given one mark each. The total score was 14 and those who scored <5 were categorized as having poor knowledge, between 6 and 10 as fair knowledge, and >10 as good knowledge. On analyzing the data, it was seen that out of those who had heard of PCOS around 60.5% showed poor knowledge. However, 14.7% had fair knowledge, and 24.9% had good knowledge regarding PCOS. When we analyzed the score categories with the education level and occupation status of the women it was seen that those who are graduates and postgraduates scored better and the results were statistically significant (P≤0.001). Those who were working had better knowledge than the rest of the participants (P≤0.001) (Tables 4-5). Lastly, we inquired about the healthy lifestyle practices followed by the study group in their day-to-day life and it was found that around 23.9% of women were doing some kind of exercise.

**Table 4:** Correlation between Education Level and Knowledge Score

Total Score	Education					Total
	ILLITERATE	PRIMARY	HIGH SCHOOL/PUC	GRADUATE	POSTGRADUATE	
0	59 (95.2%)	27 (100%)	52 (70.3%)	44 (30.8%)	8 (28.6%)	190 (56.9%)
1-5	0 (0%)	0 (0%)	4 (5.4%)	8 (5.6%)	0 (0%)	12 (3.6%)
6-10	0 (0%)	0 (0%)	7 (9.5%)	32 (22.4%)	10 (35.7%)	49 (14.7%)
11-15	3 (4.8%)	0 (0%)	11 (14.9%)	59 (41.3%)	10 (35.7%)	83 (24.9%)
Total	62 (100%)	27 (100%)	74 (100%)	143 (100%)	28 (100%)	334 (100%)
Mean ± SD	0.68±3.03	0±0	2.99±4.83	7.41±5.67	7.89±5.71	4.62±5.71

**Table 5:** Correlation of Knowledge Score and Occupation Status of the Women

Total Score	OCCUPATION			Total
	HOUSEWIFE	STUDENTS	WORKING WOMEN	
0	151 (91.5%)	16 (20.3%)	23 (25.6%)	190 (56.9%)
1-5	0 (0%)	4 (5.1%)	8 (8.9%)	12 (3.6%)
6-10	1 (0.6%)	26 (32.9%)	22 (24.4%)	49 (14.7%)
11-15	13 (7.9%)	33 (41.8%)	37 (41.1%)	83 (24.9%)
Total	165 (100%)	79 (100%)	90 (100%)	334 (100%)

Out of all 9.8% go for walk daily, 1.76% were practicing weight training, 2.69% were doing aerobic exercises, 23% were doing yoga, and 2.64% were doing swimming. Around 11.7% of the study population was practicing meditation as stress-relieving management. Around 54.6% of the women were taking healthy food items most of the time in their diet and take junk food only occasionally, however, the rest were taking junk food quite often (45.40%).

## Discussion

PCOS is a complex endocrine disorder affecting women across all stages of their life. Adolescent girls often present with menstrual irregularities, acne, hirsutism, and obesity which can further lead to body image disorders, low self-esteem, anxiety, and depression in them. Women with PCOS may have difficulty in spontaneous conception and often need fertility treatment. These women can also experience repeated pregnancy losses. Later in life, they are at increased risk of diabetes, hypertension, and cardiovascular disorders. Due to the unopposed estrogen, they are at risk of developing endometrial hyperplasia and endometrial cancer [17]. Management often includes measures targeted to reduce the symptoms or to prevent the complications associated with the disorder. There is no permanent cure but the lifestyle modifications in PCOS women like weight reduction, exercise, meditation, and a healthy diet is seen to help them to fight their symptoms. Hence it is very important to spread knowledge and awareness in the community regarding the risk factors, symptoms, complications when to seek help, and whom to seek help from. In today's world where a sedentary lifestyle, preserved food, pollution, and stressful day-to-day life have become a new norm, the importance of adopting a healthy lifestyle should be stressed upon. Several studies done previously by researchers have shown a lack of knowledge regarding the signs and symptoms of PCOS among the general population [18,19].

In the present study, researchers found that around 43.4% had heard about the term PCOS. The above findings are similar to the findings published by Alessa et al. in their study where researchers found that 56.7% of the women had heard about PCOS [20]. A significant percentage of women had heard about the disorder but still, awareness is not 100%, and a lot of efforts are required to disseminate this knowledge in the community. The most common source of information in our study was health care provider/doctor (26.6%) followed by the internet (6.28%), teacher (5.6%), and friends (4.7%), and the results are similar to studies were done by Abu-Taha M et al. and Alshdaifat E at el. [21,22]. However, the most common source of information in the study conducted by Alessa et al. was the internet (21.3%) followed by PCOS patients (10.4%), doctors (10.8%), and books (3%). Government websites and PCOS support groups were the other mentioned sources in the study. The difference could be because in our study the study participants were those attending the Gynaecological OPD for various reasons however the study done by Alessa et al. included college students. Around 9.3% of the study population was already diagnosed to have PCOS in contrast to 15.3% of the women in the study by Alessa et al. and 28.5% in a study by Rao et al. [23]. The difference can be explained by the regional variation in the distribution and variation in the sample size of the studies. In the present study, researchers found that the women who were not diagnosed with PCOS also had certain symptoms which need further evaluation. Around 35.6% reported a change in their cycle length, 9.0% had excessive hair fall, 8.7% had acne, 6.6% had a problem with conception, 6.3% reported weight gain, and 2.4% had excessive hair growth. Rao et al. in their study also found that around 40.5% of women not formally diagnosed with PCOS had PCOS-like symptoms. While assessing knowledge regarding risk factors associated with PCOS it was found that around 35% knew unhealthy eating habits can be related to PCOS while 33.3% were aware that excess weight is associated with PCOS. Around 32% said it is familial. When asked about complications related to PCOS it is seen that similar to the study by Alessa et al. in the present study participants are aware of its association with subfertility (40% vs 39%), psychological disturbances (37.1% vs 34.1%), endometrial cancer (35.9% vs 30.2%) and diabetes (28.7% vs 14.5%). Both studies found that the participants were aware that exercise and losing weight can help reduce the symptoms and related complications (37.1% vs 39.9%). Around 41% of the women were aware that eating a healthy and balanced diet is helpful and the findings are similar to the finding in the study by Alessa et al. where 34.2% were aware of the benefits of eating healthy food. In the present study around 34% of women said that medications and surgery are the other options to manage PCOS while in a study by Alessa et al. around 29.4% of women were aware of the medical management. In the study done by Alessa et al., the author has not mentioned the awareness of surgical procedures for PCOS. The present study shows education level and working status of the women were significantly associated with high knowledge scores among the study participants and the results are similar to the studies done by Alessa et al. and Alshdaifat E at el.

With increasing education levels the percentage of women who know about the risk factors, symptoms, complications, and management is also rising across the globe but is this knowledge getting translated into actual practice? To assess that we asked the study participants what health lifestyle practices they are following. In this study, only 23.95% of the study participants were exercising daily, and only 11.7% were practicing meditation. However, a significant proportion of women were taking a healthy diet and avoiding junk food (54.60%). These findings suggest that only disseminated knowledge and awareness are not enough it requires a tremendous effort to bring about behavioral changes at the community level. To bring about these behavioral changes authors suggest that knowledge and awareness about a healthy lifestyle need to be incorporated right from childhood so that they become an integral part of our lives. Behavioral modification can start from home where the parents need to be educated, schools where teachers need to be educated to disseminate the knowledge, and then social media, the internet, government websites, and television advertisements can play an important role. At community levels, teams of volunteers can arrange nuked Natak and stage shows. Hospitals and support groups should provide educational material and can arrange talks in the outpatient area of the hospital or in the community regarding PCOS awareness and the benefits of lifestyle modifications for PCOS women.

Limitations of the study

This study is done in the hospital setting so the study population is not the true representative of the general population. The knowledge regarding PCOS is assessed only in female participants and male partners are not included. The sample size is also small so the findings cannot be generalized to the whole population.

## Conclusions

PCOS is an endocrine disorder affecting women at every stage of their life. The presentation of the disorder is varied and if not diagnosed earlier can lead to dreadful long-term sequelae. No definitive management is available. The management is targeted to treat the immediate symptoms and reduce the risk of long-term sequelae. Lifestyle modifications in terms of an active lifestyle and healthy dietary habits are the first line of management and can significantly reduce the symptoms and morbidity related to the disorder. Since habits are difficult to change, hence incorporating these healthy habits early in life is vital. Despite the increasing prevalence of PCOS globally the knowledge and awareness related to the disease remain poor among the general population and calls for measures to increase community-level educational programs. Spreading awareness regarding PCOS, its risk factors, symptoms, and complications, and adherence to a healthy lifestyle is essential to reduce the burden of disease and related complications. Education level and working status are seen to be directly associated with better knowledge.

## Appendices

Annexure I: Questionnaire

Name (Optional):

1. Age:

2. Weight:

3. Height:         BMI:

4. Age of menarche:

5. Educational Qualification: 

6. Occupation: 

7. Marital Status:

Question 8. What is your cycle length:

1. <21 days

2. 21-35 days

3. 35-60 days

4. >60 days

5. Invariable

Question 9. Any change in cycle recently?

1. Yes

2. No

Question 10. If Yes? what changes?

1. Frequent cycles

2. Delayed cycles

3. Scanty periods

4. Heavy flow

5. Others (Specify)

Question 11. Have you taken any treatment for menstrual irregularity in the past 6 months?

1. Yes

2. No

Question 12. Have you experienced any weight changes recently?

1. Yes

2. No

Question 13. If Yes, what changes?

1. Weight gain

2. Weight loss

3. No Change

Question 14. Have you ever taken any treatment for excessive hair growth in the body in the past 5 years?

1. Yes

2. No

Question 15. Did you need to visit a dermatologist for excessive hair loss in the past 6 months?

1. Yes

2. No

Question 16. Did you need to visit a dermatologist for any excessive acne not responding to home remedies?

1. Yes

2. No

Question 17. Are you trying for pregnancy?

1. Yes

2. No

Question 18. If yes is there any problem in conception for which you are seeking or planning to seek treatment?

1. Yes

2. No

Question 19. Have you heard about PCOS?

 1. Yes

 2. No

Question 20. Have you been diagnosed with PCOS?

1. Yes

2. No

Question 21. From where did you hear about PCOS?

1. Teacher

2. Doctor

3. Friend

4. TV

5. Newspaper

6. Others

Question 22. Who do you think is more prone to PCOS?

1. Overweight

2. Underweight

3. Normal weight

4. Any weight

Question 23. Do you think eating unhealthy and junk food is associated with PCOS?

1. Yes

2. No

Question 24. Do you think PCOS has any problem in future childbearing?

1. Yes

2. No

Question 25. Do you think PCOS can lead to pregnancy loss?

1. Yes

2. No

Question 26. Do you have PCOS and can have any risk of deranged sugars or diabetes in the future?

1. Yes

2. No

Question 27. Do you think PCOS can have the risk of hypertension in the future?

1. Yes

2. No

Question 28. Do you think PCOS can be associated risk Cardiovascular Disorder?

1. Yes

2. No

Question 29. Do you think PCOS can be associated risk of cancer?

1. Yes

2. No

Question 30. Do you think PCOS can lead to psychological problems like depression, anxiety, and sleep disturbances?

1. Yes

2. No

Question 31. Do you think weight-changing management can help women with PCOS?

1. Yes

2. No

Question 32. Do you think exercise can help in PCOS?

1. Yes

2. No

Question 33. Do you think eating healthy and avoiding junk food can help women with PCOS?

1. Yes

2. No

Question 34. Do you think stress-relieving activities can help women with PCOS?

1. Yes

2. No

Question 35. Do you think medication is the only cure for PCOS?

1. Yes

2. No

Question 36. Do you think Surgery is the only cure?

1. Yes

2. No

Question 37. Do you exercise regularly?

1. Yes

2. No

Question 38. What kind of exercise do you do?

1. Regular walk

2. Swimming

3. Aerobic

4. Weight Training

5. Yoga

7. Other

6. None

Question 39. Do you practice any kind of meditation?

1. Yes

2. No

Question 40. How often do you eat junk food?

1. Very Often

2. Sometimes

3. Rarely
